# Supplementary material for: D2 autoreceptors gate vulnerability to cocaine use disorder
Source: bioRxiv. 2026 Mar 11:2026.03.10.710882. Preprint. [Version 1] doi: 10.64898/2026.03.10.710882 (PMC13060886; doi:10.64898/2026.03.10.710882)
Supplement: Supplement 1 [file media-1.pdf]

SUPPLEMENTAL FIGURES

D2 autoreceptors gate vulnerability to cocaine use disorder

Erin M Murray[1], Daniel Diaz-Urbina[2], Roland Bock[1,2,3], Emilya Ventriglia[3,4], Anna Tischer[4], Jung Hoon Shin[1], Seul Ah Lee[2], Lucy G Anderson[2], Sydney Cerveny[2], Isabel Bleimeister[1], Miriam E Bocarsly[1,5], Michael Michaelides[4] and Veronica A Alvarez[1,2,3,4]

1, NIAAA, National Institutes of Health, Bethesda, MD 20892
2, NIMH, National Institutes of Health, Bethesda, MD 20892
3, Center on Compulsive Behaviors, National Institutes of Health, Bethesda, MD 208923,
4, NIDA, National Institutes of Health, Baltimore, MD 21224
5, Department of Pharmacology, Physiology and Neuroscience, Rutgers New Jersey Medical School, Rutgers Brain Health Institute. NJ 08550

Supplementary Figure 1 - Murray et al.

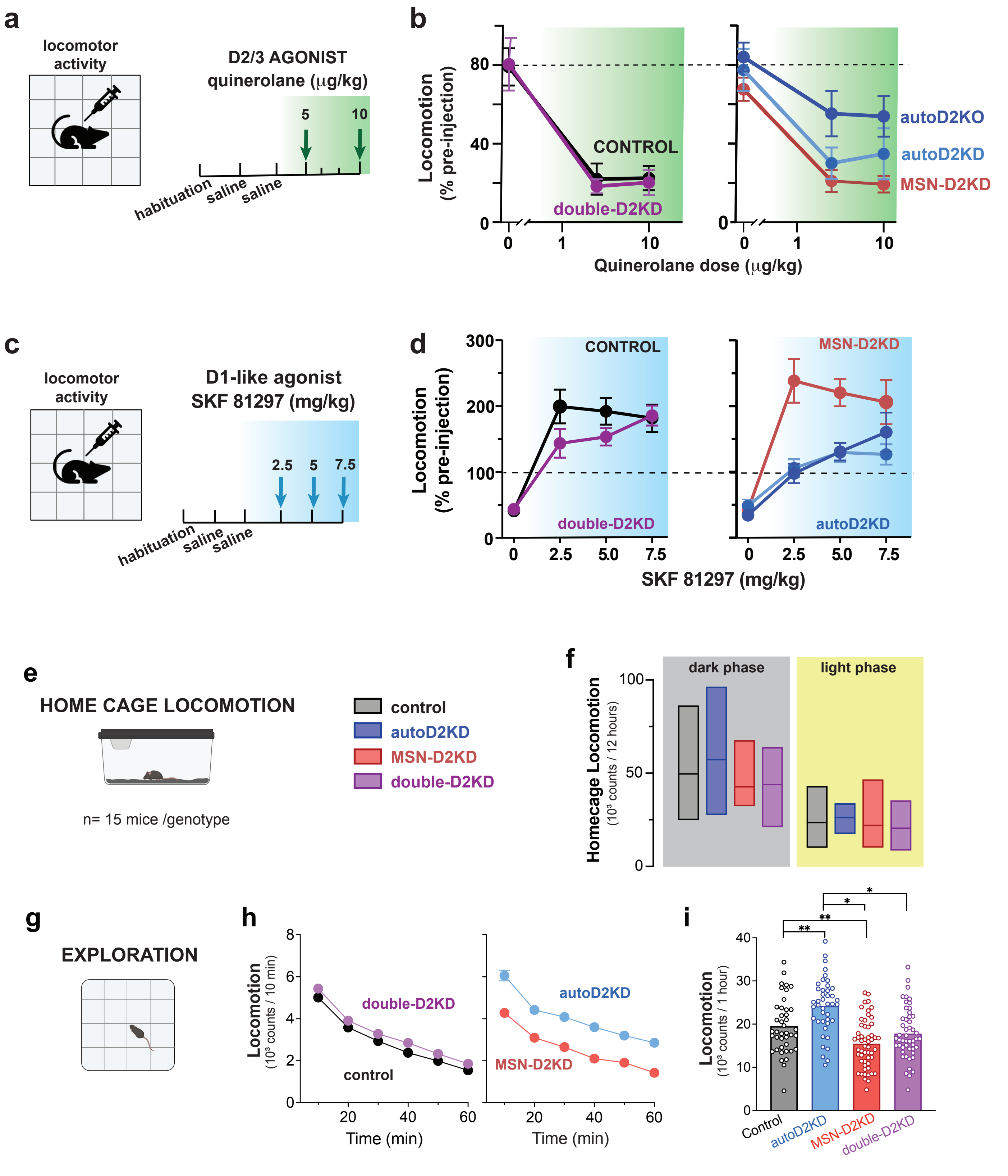

Supplementary Fig. 1. Basal locomotion and responses to dopamine agonists.

a,c, Experimental timelines for assessing dose–response effects of the D2/3 agonist quinelorane (5, 10 µg/kg, i.p.) (**a**) and the D1-like agonist SKF81297 (2.5, 5, 7.5 mg/kg, i.p.) (**c**) in locomotor activity chambers. Most mice were tested with both agonists, with a 2-week washout; SKF81297 was tested first.

b, **d**, Locomotor activity following saline or agonist injection, expressed as percent of each mouse's pre-injection baseline for quinelorane (**b**) * main effect of dose: $F(1.83,43.81) = 54.22$, $p < 0.0001$; no genotype $F(3,67) = 0.8$ $p = 0.49$; and for SKF81297(**d**), main effect of dose: $F(2.373, 178.8) = 46.98$, $p < 0.0001$; main effect of genotype: $F(4, 80) = 6.366$, $p = 0.0002$). Left plot for controls (black) and double-D2KD (purple). Right plot for autoD2KD (blue), auto-D2KO (dark blue), and MSN-D2KD (red). n = 7–15 mice per genotype per dose. Symbols indicate group means; error bars, ± s.e.m. *$p \le 0.05$ versus control.

e, Schematic of the single-housed home-cage locomotor monitoring setup.

f, Basal locomotion during the light and dark phases (n = 15 mice per genotype). Box plots show min–max; center line indicates the mean.

g, Novelty-driven exploration assessed in a novel open-field arena.

h, Time course of novelty-induced locomotion (10-min bins) for littermate mice of each genotype (n = 41 - 53).

i, Total novelty-induced locomotion over 60 min. Points show individual mice; bars indicate mean ± s.e.m. **$p \le 0.01$ versus control.

Supplementary Figure 2 - Murray et al.

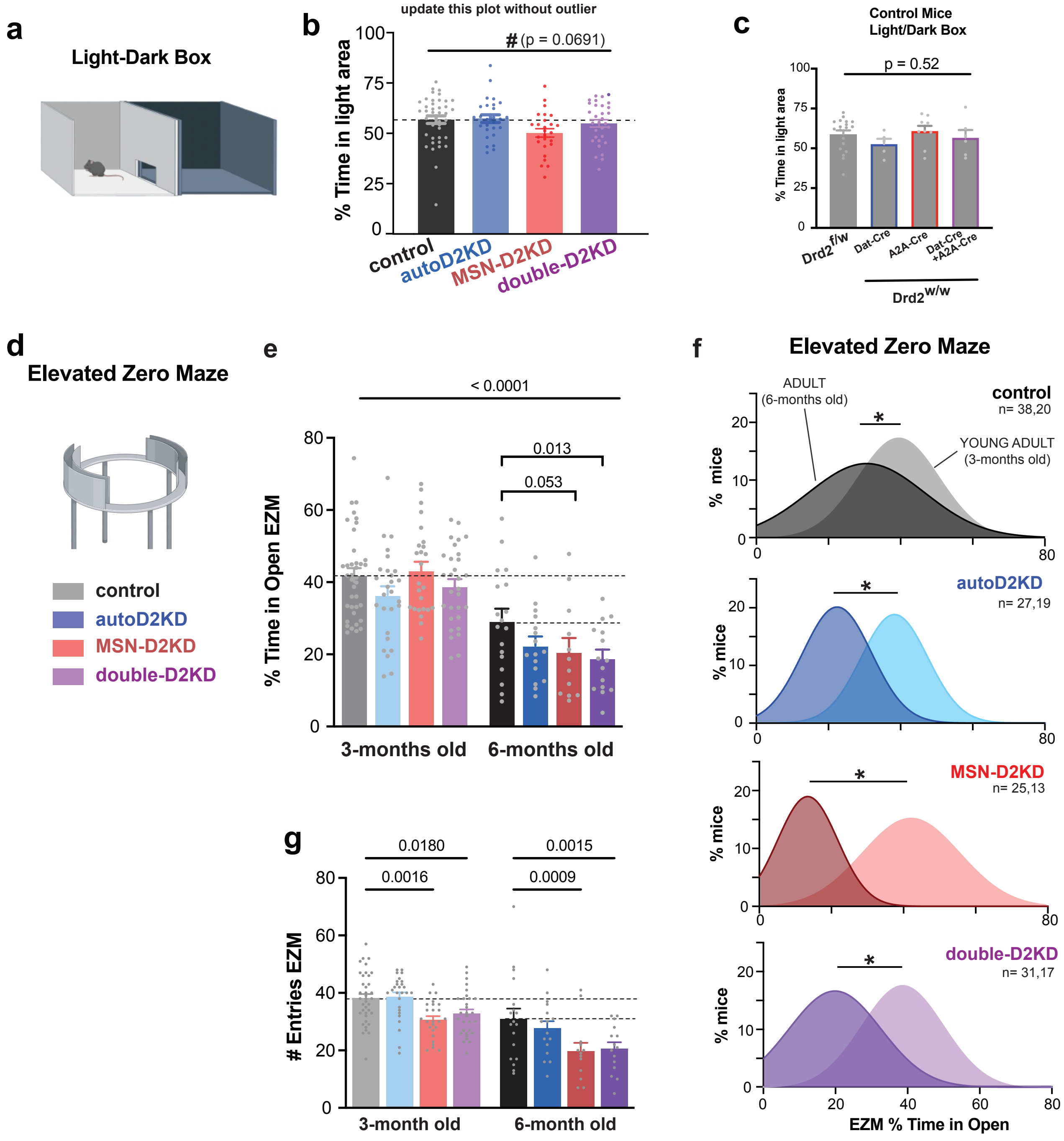

Supplementary Fig. 2. Risk-avoidance behavior.

a, Schematic of the light–dark box.

b, Percent time spent in the illuminated compartment for littermate mice of each genotype (n = 38 control, 27 autoD2KD, 25 MSN-D2KD and 31 double-D2KD). Points indicate individual mice; bars show mean ± s.e.m. #, trend for a genotype effect (one-way ANOVA, p = 0.069).

c, Light–dark box performance in Cre-driver control lines $Drd2^{f/w}$, $Drd2^{w/w}$; $DAT^{IRES\text{-}Cre}$, $Drd2^{w/w}$;$Adora2A\text{-}Cre^{+/-}$, $Drd2^{w/w}$;$DAT^{IRES\text{-}Cre}$; $Adora2a\text{-}Cre^{+/-}$ show no differences between control types (one-way ANOVA, F (3, 35) = 0.769, p = 0.518).

d, Schematic of the elevated zero maze (EZM).

e,g, EZM performance at 3 and 6 months of age: percent time in open quadrants (**e**) and number of open-quadrant entries (**g**) for each genotype (3 months / 6 months: controls n = 38 / 20; autoD2KD n = 27 / 19; MSN-D2KD n = 25 / 13; double-D2KD n = 31 / 17). Points indicate individual mice; bars show mean ± s.e.m. Two-way ANOVA main effect of age, p = 0.0001; post hoc p values are indicated.

f, Frequency distributions of time spent in the illuminated compartment in the light–dark box at 3 months (light shading) and 6 months (dark shading) of age. *p ≤ 0.05.

Supplementary Figure 3 - Murray et al.

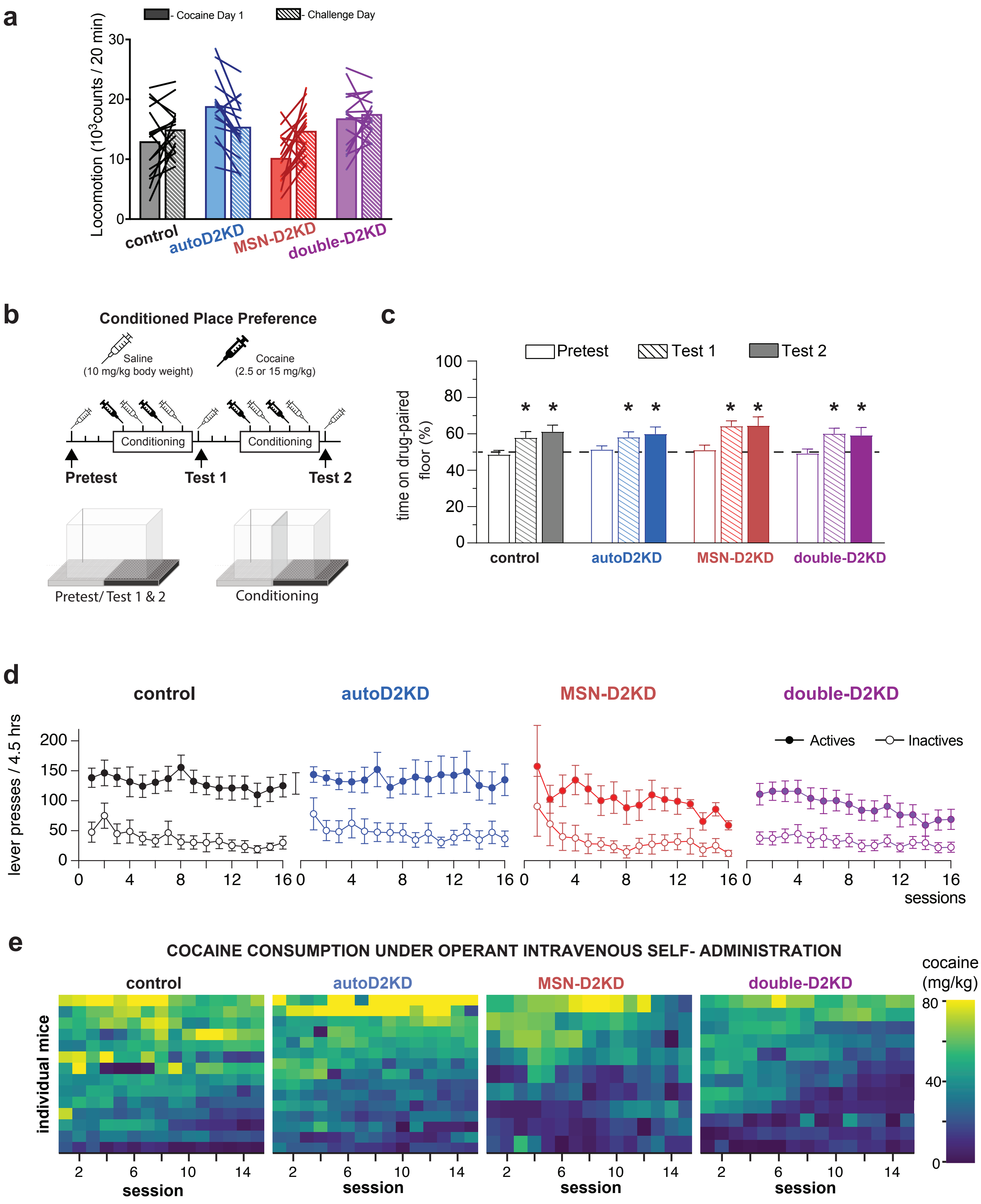

Supplementary Fig. 3. Acute and repeated cocaine responses.
a, Cocaine-induced locomotion (20-min epoch) during the first cocaine exposure (day 1) and during the cocaine challenge after 2 weeks of abstinence (controls, n = 16; autoD2KD, n = 17; MSN-D2KD, n = 15; double-D2KD, n = 15). Points show individual mice; lines connect paired measurements within mice; bars indicate mean ± s.e.m.
b, Experimental timeline and schematic of the conditioned place preference (CPP) apparatus.
c, Percentage of time spent on the cocaine-paired floor during the pre-test (open bars), Test 1 (striped bars) and Test 2 (filled bars) of conditioned place preference (n = 13-15 mice per genotype). Bars indicate mean ± s.e.m.; *$p \leq 0.05$.
d, Lever-press rates on the active (filled symbols) and inactive (open symbols) levers across sessions for each genotype (controls, n = 13; autoD2KD, n = 15; MSN-D2KD, n = 9; double-D2KD, n = 12). Symbols indicate mean ± s.e.m.
e, Heat maps of daily cocaine intake (mg kg^{-1}) across 16 IVSA sessions for individual mice in each genotype; color scale denotes intake.

Supplementary Figure 4 - Murray et al.

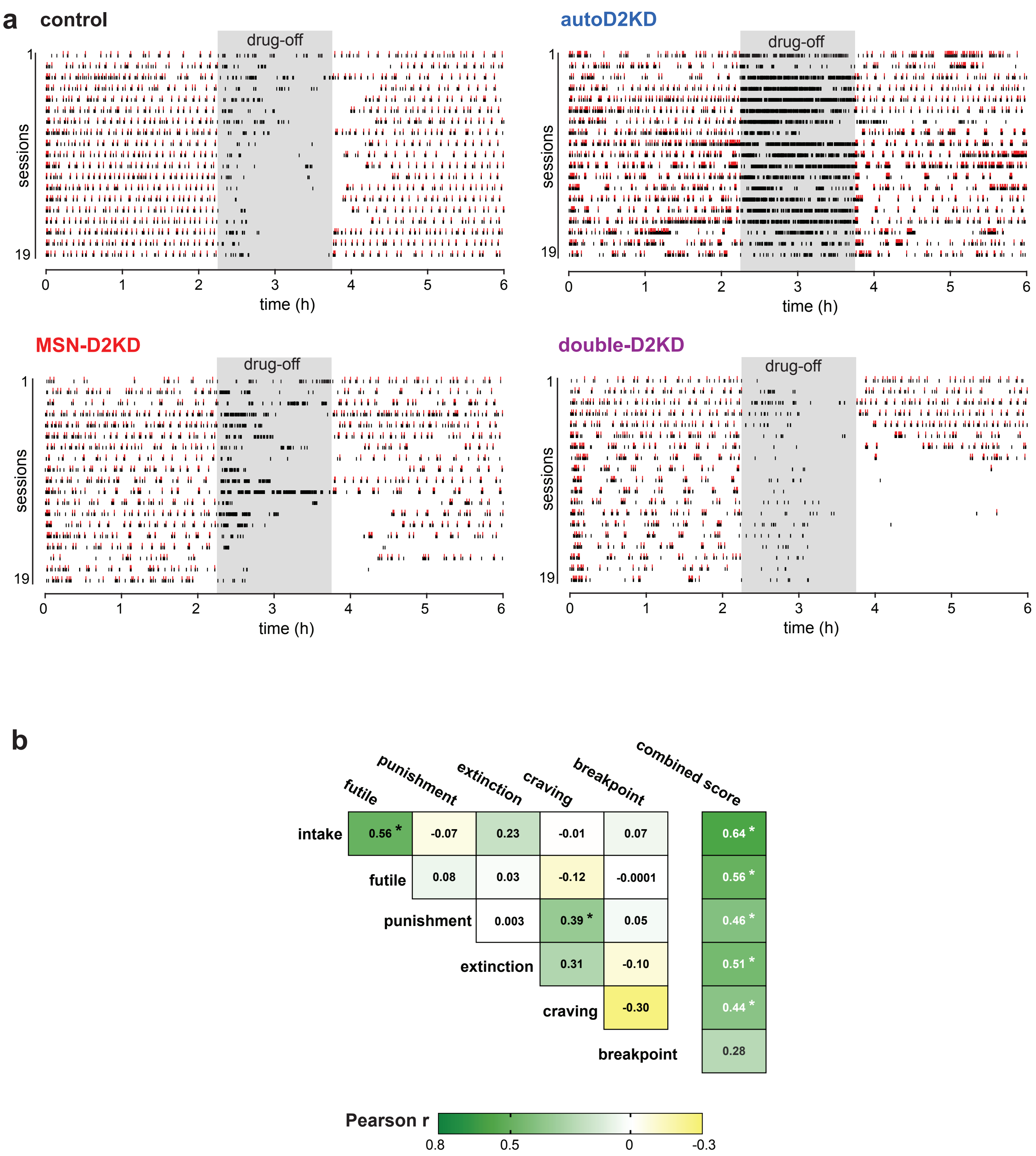

Supplementary Fig. 4. Cocaine intravenous self-administration enables multi-domain assessment of addictive-like behaviors.

a, Representative rater plots of cocaine IVSA during 19 sessions after acquisition. Black ticks indicate active-lever presses and red ticks indicate earned cocaine infusions (1 mg/kg/infusion) under FR3 schedule. Each 6-hour session included a 90 min signaled (“drug-off”) period (shaded), during which responses had no programmed consequences and were quantified as futile responding.

b, Correlation matrix across cocaine-related behavioral measures. Colors indicate Pearson’s r; * $p < 0.05$.

Supplementary Figure 5 - Murray et al.

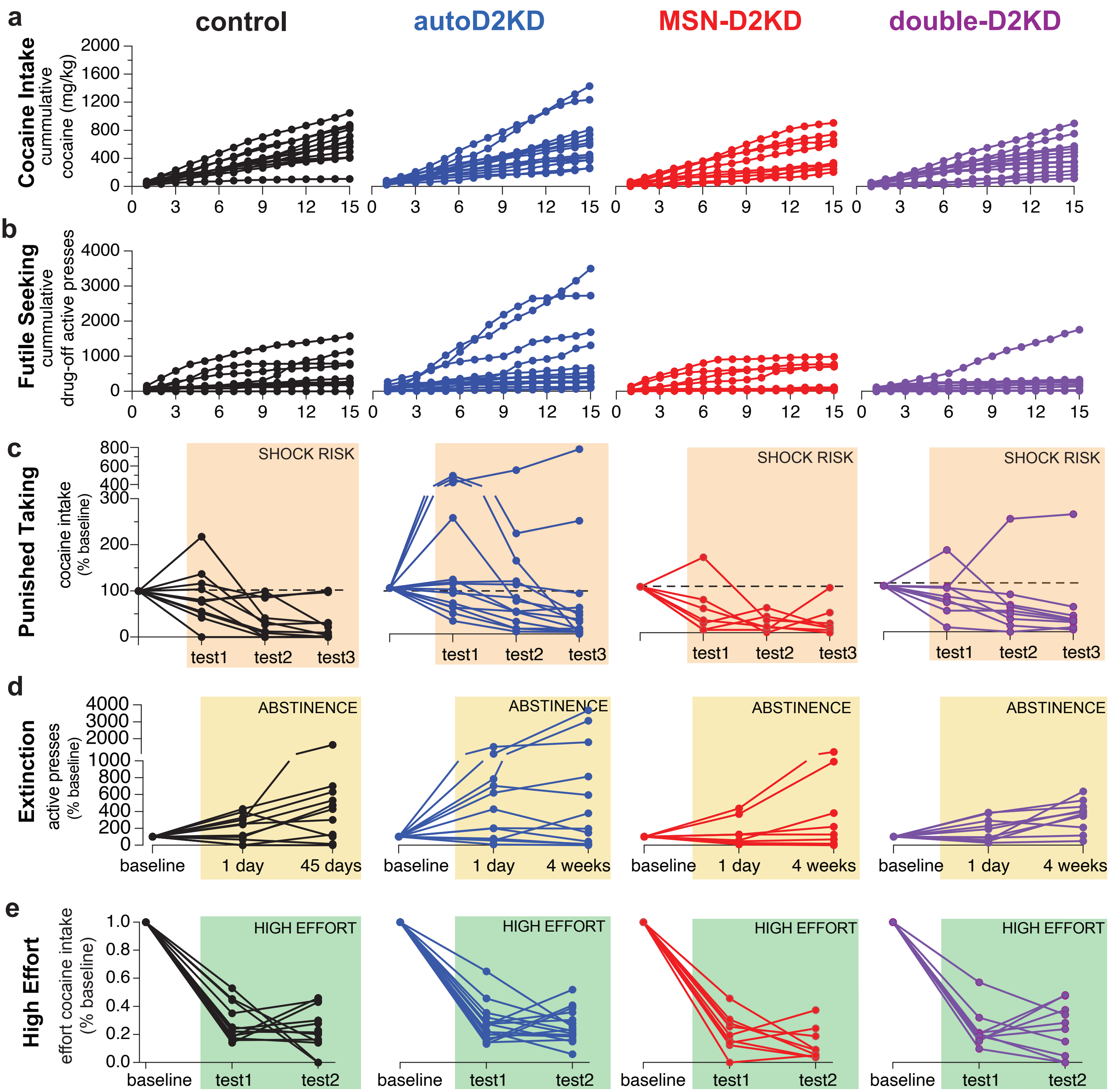

Supplementary Fig. 5. autoD2KD mice show increased inter-individual variability in cocaine-related behaviors during IVSA.

a,b, Across-session trajectories for cumulative cocaine intake (a) and futile responding during the signaled drug-unavailable (“drug-off”) period (b) over 16 IVSA sessions for controls (n = 13, black), autoD2KD (n = 15, blue), MSN-D2KD (n = 9, red) and double-D2KD (n = 12, purple). Symbols indicate individual mice.

c–e, Individual performance during foot-shock punishment across three tests (c), cue/context-driven seeking during abstinence (d), and high-effort responding assessed with progressive-ratio tests (e). Data are normalized to each mouse’s baseline (pre-punishment intake, pre-abstinence responding, or FR3 performance, respectively).

Supplementary Figure 6- Murray et al.

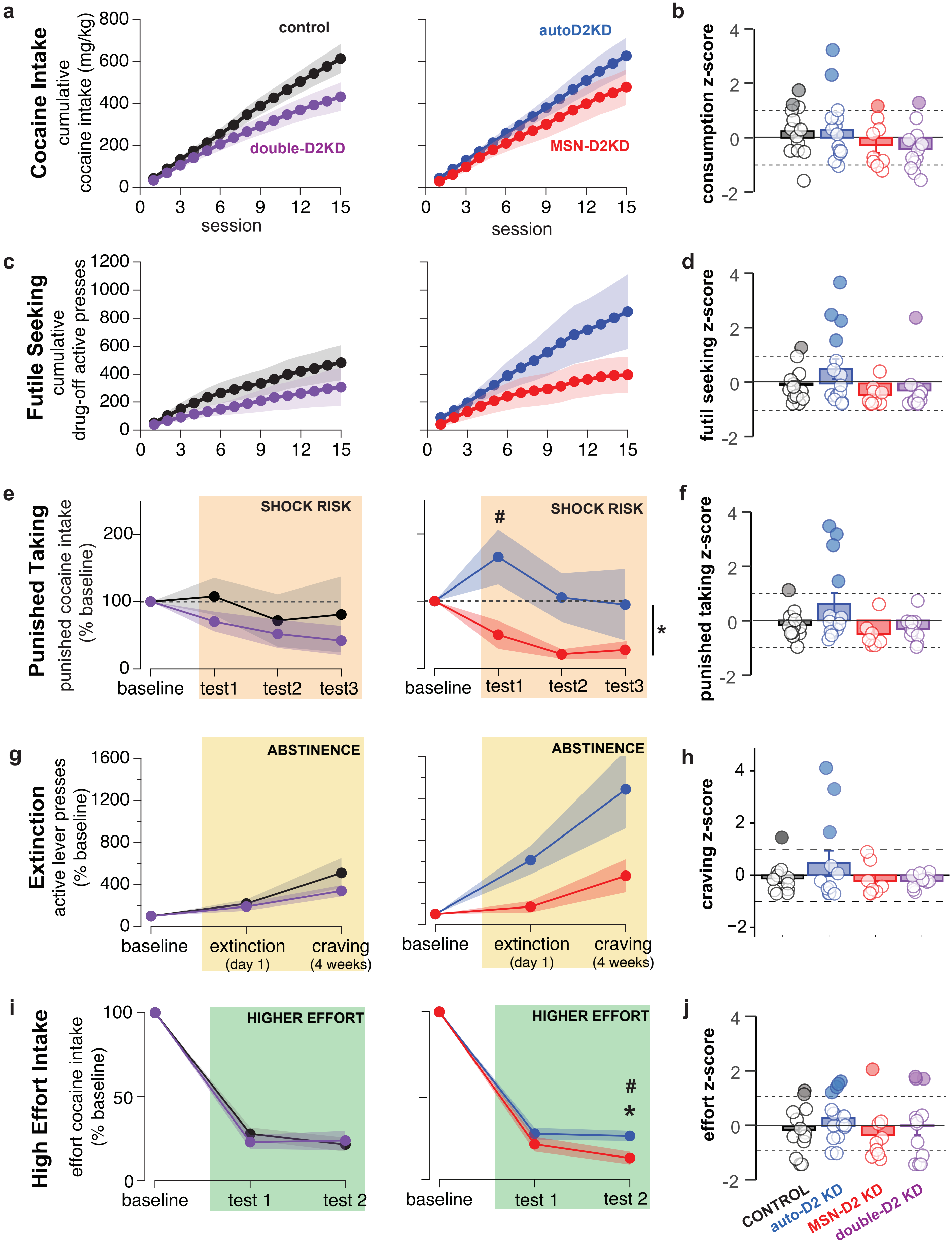

Supplementary Fig. 6. Cocaine IVSA behavioral measures and z-score across genotypes. **a,c,e,g,i,** Mean behavioral measures by genotype for cumulative cocaine intake (**a**), cumulative futile responding (**c**), punished cocaine consumption (**e**), extinction and craving responding (**g**), and high-effort consumption (**i**). For each measure, the left panel shows controls (black) and double-D2KD (purple), and the right panel shows autoD2KD (blue) and MSN-D2KD (red). Symbols indicate group means; shaded bands show ± s.e.m.

b,d,f,h,j, Z-scores by genotype for cocaine consumption (**b**), futile seeking (**d**), punished cocaine consumption (**f**), craving (**h**), and high-effort intake (**j**). Points indicate individual mice; bars show mean.

For all panels, *P ≤ 0.05 and #0.05 < P ≤ 0.1 versus control.

Supplementary Figure 7- Murray et al.

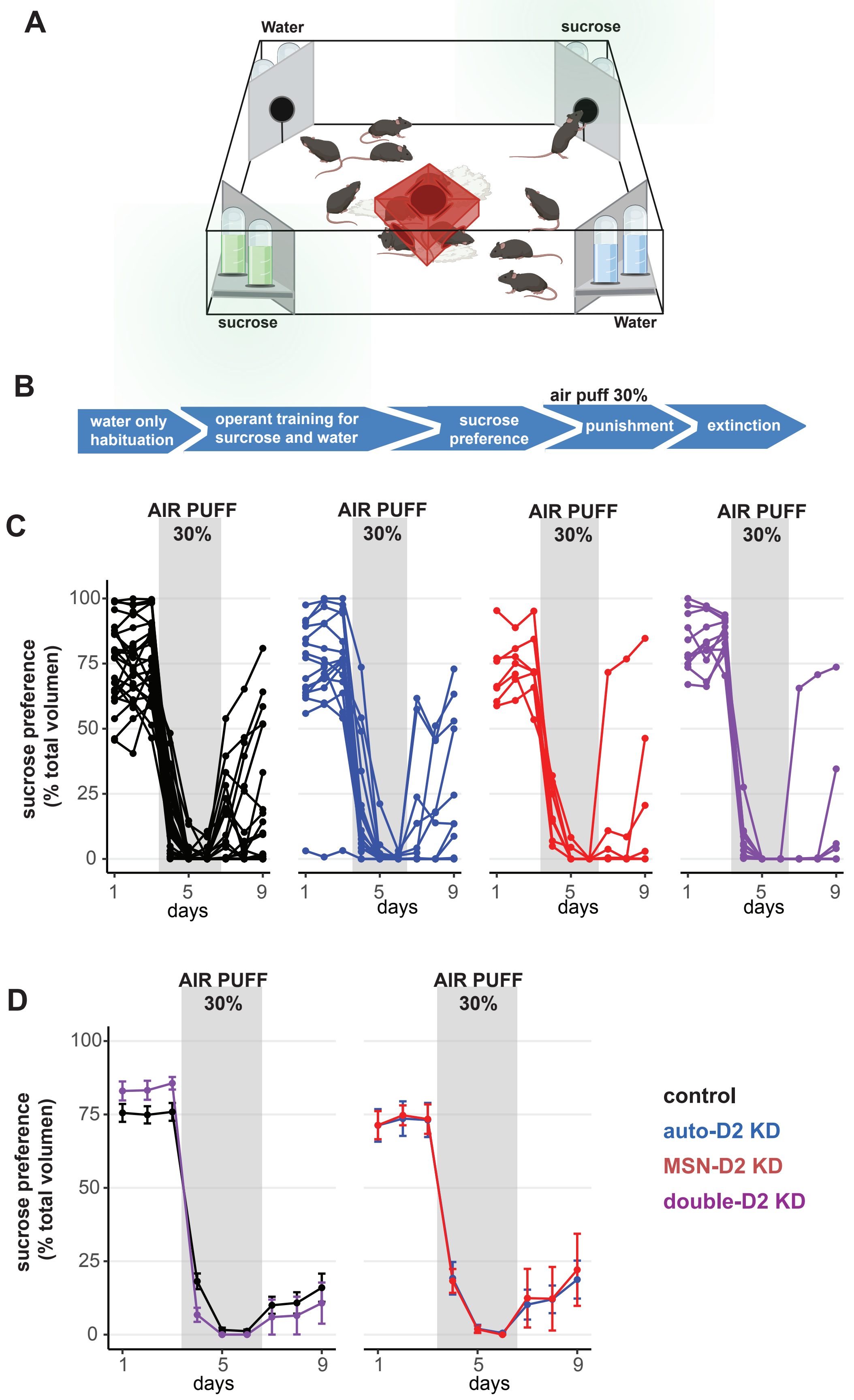

Supplementary Fig. 7. Intact sucrose preference and punishment sensitivity across genotypes

a, Schematic of the IntelliCage system for self-paced operant testing in socially housed mice. **b,** Experimental timeline showing operant access to water and 1% sucrose, followed by a 3-day punishment phase in which 30% of sucrose-access events were paired with an air puff (2 s, psi). **c,d,** Sucrose preference (sucrose licks / water licks) shown for individual mice (**c**) and as genotype means (**d**) for controls (black), autoD2KD (blue), MSN-D2KD (red) and double-D2KD (purple).
