# Supplementary material for: D2 autoreceptors gate vulnerability to cocaine use disorder
Source: bioRxiv. 2026 Mar 11:2026.03.10.710882. Preprint. [Version 1] doi: 10.64898/2026.03.10.710882 (PMC13060886; doi:10.64898/2026.03.10.710882)
Supplement: Supplement 2 [file media-2.pdf]

Murray et al. Statistical Anlaysis Data Table File

D2 autoreceptors gate vulnerability 1 to cocaine use disorder

Figure	Measurement	# of mice	Statistical test	Results	p value
			**Region**		
			Interaction (Brain Region x Genotype)	F (6, 28) = 1.249	0.3121
			Region	F (1.247, 17.46) = 5.341	0.027
			Genotype	F (3, 16) = 12.81	0.0002
			**Dunnett's multiple comparisons test (region)**		
			Cortex		
			Control vs. double-D2KD		0.7593
			Control vs. Auto-D2KD		0.9496
1b	Ddr2 mRNA expression	n = 3 - 5	Control vs. MSN-D2KD		0.6129
			NAc		
			Control vs. double-D2KD		0.021
			Control vs. Auto-D2KD		0.418
			Control vs. MSN-D2KD		0.0192
			Dorsal Striatum		
			Control vs. double-D2KD		0.001
			Control vs. Auto-D2KD		0.3019
			Control vs. MSN-D2KD		0.0002
			**Region**		
			Interaction (Brain Region x Genotype)	F (3, 32) = 5.865	0.0026
			Region	F (1, 32) = 11.11	0.0022
			Genotype	F (3, 32) = 43.36	<0.0001
			**Dunnett's multiple comparisons test (region)**		
			Dorsal Striatum		
1d	D2-like receptor radioligand binding	n = 3 - 9	Control vs. double-D2KD		<0.0001
			Control vs. Auto-D2KD		0.0041
			Control vs. MSN-D2KD		<0.0001
			NAc		
			Control vs. double-D2KD		0.0037
			Control vs. Auto-D2KD		0.064
			Control vs. MSN-D2KD		<0.0001
			**2W ANOVA - Genotype x Region**		
			Interaction (Genotype x Region)	F (3, 12) = 5.122	0.0165

			Genotype	F (3, 12) = 5.995	0.0098
			Region	F (1, 12) = 10.74	0.0066
			**Tukey's multiple comparisons test (region)**		
			Dorsal Striatum		
			double-D2KD vs. AutoD2		0.984
			AutoD2 vs. Control		0.8211
			AutoD2 vs. MSN		0.1082
1f	D1-like receptor radioligand binding	n = 3 - 6	double-D2KD vs. Control		0.5839
			double-D2KD vs. MSN		0.0496
			MSN vs. Control		0.269
			NAc		
			double-D2KD vs. AutoD2		0.7971
			AutoD2 vs. Control		0.0183
			AutoD2 vs. MSN		0.0006
			double-D2KD vs. Control		0.1653
			double-D2KD vs. MSN		0.007
			MSN vs. Control		0.2445

sig?
*
***
ns
ns
ns
*
ns
*
***
ns
***
**
**
***
****
**
****
**
#
****
*

**
**
ns
ns
ns
ns
*
ns
ns
*
***
ns
**
ns

Figure	Measurement	# of mice	Statistical test
2b	D1/D2 Receptor Ratio (Radioligand binding)	n = 3 - 6	**2W mixed-effects model - Genotype x [illegible]**<br>Interaction (Region x Genotype)<br>Region<br>Genotype<br>**Dunnett's multiple comparisons test**<br>*Dorsal Striatum*<br>Control vs. double-D2KD<br>Control vs. Auto-D2KD<br>Control vs. MSN-D2KD<br>*NAc*<br>Control vs. double-D2KD<br>Control vs. Auto-D2KD<br>Control vs. MSN-D2KD
2d	Locomotor Response to 5 µg/kg Quinelorane (D2-like agonist)	n = 6 - 11	**One-way ANOVA**
2d	D1-like (5 mg/kg) and D2/3-like (5 ug/mg) agonist locomotor response	n = 6 - 21	**2W ANOVA - Genotype x Agonist Type**<br>Interaction (Genotype x Agonist Type)<br>Genotype<br>Agonist Type<br>**Dunnett's multiple comparisons test**<br>*D2/3-like agonist (quinelorane)*<br>Control vs. Auto-D2KD<br>Control vs. MSN-D2KD<br>Control vs. double-D2KD<br>*D1-like agonist (SKF81297)*<br>Control vs. Auto-D2KD<br>Control vs. MSN-D2KD<br>Control vs. double-D2KD

Results	p value	Significance?
F (3, 25) = 7.258	0.0012	**
F (1, 25) = 0.2645	0.6115	ns
F (3, 25) = 22.74	<0.0001	****
	<0.0001	****
	0.4828	ns
	<0.0001	****
	0.8612	ns
	0.7579	ns
	0.0002	***
F (3, 31) = 0.6602	0.5828	ns
F (3, 97) = 2.894	0.0392	*
F (3, 97) = 2.020	0.1162	ns
F (1, 97) = 56.21	<0.0001	****
	0.9869	ns
	0.9997	ns
	>0.9999	ns
	0.0165	*
	0.4409	ns
	0.1879	ns

Figure	Measurement	# of mice	Statistical test	Results	p value	Significance?

Figure	Measurement
4c, S1h-i	Open Field Locomotion: Novel Exploration
4d, S2b	Light-Dark Box: Duration in Light Area

# of mice	Statistical test
	**One-way ANOVA**
	**Tukey's multiple comparisons test**
n = 41 - 53	DbHET vs. AutoD2
	DbHET vs. MSN-D2KD
	DbHET vs. Control
	AutoD2 vs. MSN-D2KD
	AutoD2 vs. Control
	MSN-D2KD vs. Control
	**One-way ANOVA**
	**Dunnett's multiple comparisons test**
n = 25 - 38	Control vs. double-D2KD
	Control vs. Auto-D2KD
	Control vs. MSN-D2KD

Results	p value	Significance?
F (3, 183) = 17.28	<0.0001	****
	<0.0001	****
	0.2077	ns
	0.5304	ns
	<0.0001	****
	0.0029	**
	0.0079	**
F (3, 119) = 3.32		
	0.5116	ns
	0.9924	ns
	0.0107	*

Figure	Measurement	# of mice
5b	Cocaine Sensitization: Habitutation Locomotor Response	n = 16 - 18
5b	Cocaine Sensitization: comparisons across saline day 1 through cocaine day 5	n = 16 - 18

5c	Cocaine Sensitization: Comparison of Cocaine Sensitization Score	n = 16 - 18

Statistical test	Results	p value	?
**One-way ANOVA**	F (3, 63) = 5.281	0.0026	**
**Dunnett's multiple comparisons test**			
Control vs. Auto-D2KD		0.2863	ns
Control vs. MSN-D2KD		0.0746	ns
Control vs. double-D2KD		0.5735	ns
**2W mixed-effects model - Session Day x Genotype**			
Interaction (Cocaine x Genotype)	F (15.32, 317.5) = 3.874	<0.0001	****
Session Day	F (5.107, 317.5) = 117.6	<0.0001	****
Genotype	F (3, 63) = 6.183	0.0009	***
**Dunnett's multiple comparisons test**			
Saline Day			
Control vs. double-D2KD		0.8983	ns
Control vs. Auto-D2KD		0.0508	ns
Control vs. MSN-D2KD		0.8562	ns
Cocaine Day 1			
Control vs. double-D2KD		0.1286	ns
Control vs. Auto-D2KD		0.0123	*
Control vs. MSN-D2KD		0.4152	ns
Cocaine Day 2			
Control vs. double-D2KD		0.0913	ns
Control vs. Auto-D2KD		0.0076	**
Control vs. MSN-D2KD		0.8944	ns
Cocaine Day 3			
Control vs. double-D2KD		0.0243	*
Control vs. Auto-D2KD		0.7034	ns
Control vs. MSN-D2KD		0.8636	ns
Cocaine Day 4			
Control vs. double-D2KD		0.0092	**
Control vs. Auto-D2KD		0.4477	ns
Control vs. MSN-D2KD		0.9963	ns
Cocaine Day 5			
Control vs. double-D2KD		0.0355	*

Control vs. Auto-D2KD		0.4497	ns
Control vs. MSN-D2KD		0.7022	ns
Challenge Day			
Control vs. double-D2KD		0.0768	ns
Control vs. Auto-D2KD		0.9813	ns
Control vs. MSN-D2KD		0.861	ns
**One-way ANOVA**	F (3, 61) = 4.45	0.0068	**
**Tukey's multiple comparisons test**			
double-D2KD vs. Auto-D2KD		0.3533	ns
double-D2KD vs. MSN-D2KD		0.4339	ns
Control vs. double-D2KD		0.4633	ns
Auto vs. MSN-D2KD		0.0141	*
Control vs. Auto-D2KD		0.015	*
Control vs. MSN-D2KD		0.9999	ns

Figure	Measurement	# of mice	Statistical test
6c	IVSA: genotype rate of aquisition	n = 9 - 15	**Chi-Square**
6d	IVSA: days to task aquisition	n = 9 - 15	**One-way ANOVA**
6f	IVSA: cummulative cocaine intake		**Two-way RM ANOVA**
			sessions x Genotype
		n = 13 - 22	sessions
			Genotype
6g	IVSA: total cocaine consumption	n = 13 - 22	**Unpaired T-test**
6h	IVSA: cummulative futile responses	n = 13 - 22	**Two-way RM ANOVA**
			sessions x Genotype
			sessions
			Genotype
			Subject
6i	IVSA: total futile responses	n = 13 - 22	**One-way ANOVA**
			autoD2KD vs MSN-D2KDcombined
6j	IVSA: punished consumption	n = 13 - 22	**Two-way RM ANOVA**
			Punishment
			Genotype
			Punishment x Genotype
6k	IVSA: first punishment test	n = 13 - 22	**One-way ANOVA**
			autoD2KD vs MSN-D2KDcombined
6l	IVSA: extinction and craving	n = 13 - 22	**Two-way RM ANOVA**
			Abstinence x Genotype
			Abstinence
			Genotype
			Subject
6m	IVSA: extinction	n = 13 - 22	**One-way ANOVA**
			autoD2KD vs MSN-D2KDcombined
			control vs autoD2KD
6n	IVSA: high effort consumption	n = 13 - 22	**Two-way RM ANOVA**
			High Effort x Genotype
			High Effort
			Genotype
			Subject
6o	IVSA: z-scores	n = 13 - 22	**Mixed-effects model (REML)**
			Genotypes
			Behaviors
			Genotypes x Behaviors

Results	p value	?
X² = 0.24	0.97	ns
(3, 45) = 0.215	0.8852	ns
F (28, 644) = 1.[illegible]	P=0.0036	Yes
F (1.120, 51.53[illegible]	P<0.0001	Yes
F (2, 46) = 1.91	P=0.1595	ns
t (34) = 1.8	0.075	#
F (28, 672) = 2.[illegible]	P=0.0012	yes
F (1.149, 55.14[illegible]	P<0.0001	yes
F (2, 48) = 2.81[illegible]	P=0.0700	#
F (48, 672) = 3[illegible]	P<0.0001	yes
F (2, 48) = 2.65[illegible]	P=0.0803	#
post hoc	P=0.0741	#
F (1.777, 74.64[illegible]	0.0365	yes
F (2, 43) = 2.84[illegible]	0.0693	#
F (4, 84) = 2.16[illegible]	0.0797	#
F (2, 42) = 3.77[illegible]	P=0.0311	yes
post hoc	P=0.0234	yes
F (4, 76) = 1.65	P=0.1702	ns
F (1.155, 43.87[illegible]	P=0.0004	yes
F (2, 38) = 2.83[illegible]	P=0.0715	#
F (38, 76) = 1.9[illegible]	P=0.0064	yes
F (2, 38) = 4.68[illegible]	P=0.0151	yes
post hoc	P=0.0153	yes
post hoc	P=0.0656	#
F (4, 86) = 0.62[illegible]	P=0.6447	ns
F (2, 86) = 650.[illegible]	P<0.0001	yes
F (2, 43) = 2.06	P=0.1397	ns
F (43, 86) = 0.8[illegible]	P=0.6620	ns
F (2, 46) = 7.38[illegible]	0.0017	yes
F (3.620, 151.3[illegible]	0.8154	ns
F (10, 209) = 1.[illegible]	0.4089	ns

Figure	Measurement	# of mice	Statistical test	Results
7a	IVSA: genotype rate of aquisition	n = 13,15,21	**Two-way ANOVA**	
			Interaction	F (10, 264) = 0.6293
			Behaviors	F (5, 264) = 0.02231
			Genotype	F (2, 264) = 7.624
7b	Addictive like Behaviors: z-scores	n = 13,15, 21	**One-way ANOVA**	F (2, 46) = 6.872
			control vs. autoD2KD	
			control vs. MSN-D2KDcombined	
			autoD2KD vs. MSN-D2KDcombined	
7c	Addictive like Behaviors: Proportions	n = 13, 15, 21	**Fisher's exact test**	

p value	?
0.7883	ns
0.9998	ns
0.0006	yes
0.0024	yes
0.0688	#
0.5315	ns
0.0017	yes
0.007	Yes

Figure	Measurement	# of mice
S1b	Controls Locomotor Dose Response to D2-like agonist (Quinelorane): 0 , 5 and 10 µg/kg	n = 6 - 15
S1b	Control vs. AutoD2-KO Locomotor Dose Response to D2-like agonist (Quinelorane): 5 and 10 µg/kg	n = 6 - 16
S1b	Locomotor Dose Response to D2-like agonist (Quinelorane): 0, 5 and 10 µg/kg	n = 6 - 16

S2d

Locomotor Dose Response to D1-like Agonist (SKF81297): 0, 2.5, 5.0, and 7.5 mg/kg

n = 15 - 21

SF 1f	Open Field Locomotion: 24-Hour Homecage Baseline	n = 45, 15 per genotype

Statistical test	Results	p value	Significance?
**Mixed-effects analysis**	F (1.954, 8.794) = 12.46	0.0028	**
**Two-way RM ANOVA**			
Interaction (Genotype x Dose)	F (1, 18) = 0.01359	0.9085	ns
Dosage	F (1, 18) = 0.003238	0.9552	ns
Genotype	F (1, 18) = 4.341	0.0517	#
**Mixed-effects analysis**			
Interaction (Genotype x Dose)	F (5.476, 43.81) = 0.1983	0.969	ns
Dosage	F (1.825, 43.81) = 54.22	<0.0001	****
Genotype	F (3, 67) = 0.8041	0.496	ns
**Tukey's multiple comparisons test (region)**			
0 µg/kg (saline) vs. 0.005 µg/kg		<0.0001	****
0 µg/kg vs. 0.01 µg/kg		<0.0001	****
0.005 µg/kg vs. 0.01 µg/kg		0.9265	ns
**2W mixed-effects model - Genotype x D1-Agonist Dose**			
Interaction (Genotype x Dose)	F (12, 226) = 2.000	0.0253	*
Dosage	F (2.373, 178.8) = 46.98	<0.0001	****
Genotype	F (4, 80) = 6.366	0.0002	***
**Dunnett's multiple comparisons test (dosage)**			
_0_			
double-D2KD vs. AutoD2		0.9937	ns
double-D2KD vs. MSN		>0.9999	ns
double-D2KD vs. Control		0.9993	ns
double-D2KD vs. Auto-D2KO		0.8541	ns
AutoD2 vs. MSN		0.9789	ns
AutoD2 vs. Control		0.976	ns
AutoD2 vs. Auto-D2KO		0.7254	ns
MSN vs. Control		0.9999	ns
MSN vs. Auto-D2KO		0.8148	ns
Control vs. Auto-D2KO		0.9671	ns

2.5		
double-D2KD vs. AutoD2	0.5877	ns
double-D2KD vs. MSN	0.1419	ns
double-D2KD vs. Control	0.4711	ns
double-D2KD vs. Auto-D2KO	0.4271	ns
AutoD2 vs. MSN	0.0081	**
AutoD2 vs. Control	0.0281	*
AutoD2 vs. Auto-D2KO	0.9946	ns
MSN vs. Control	0.8834	ns
MSN vs. Auto-D2KO	0.0051	**
Control vs. Auto-D2KO	0.0171	*
5		
double-D2KD vs. AutoD2	0.7529	ns
double-D2KD vs. MSN	0.0688	#
double-D2KD vs. Control	0.5101	ns
double-D2KD vs. Auto-D2KO	0.7478	ns
AutoD2 vs. MSN	0.0093	**
AutoD2 vs. Control	0.1253	ns
AutoD2 vs. Auto-D2KO	>0.9999	ns
MSN vs. Control	0.8627	ns
MSN vs. Auto-D2KO	0.0079	**
Control vs. Auto-D2KO	0.1175	ns
7.5		
double-D2KD vs. AutoD2	0.0738	#
double-D2KD vs. MSN	0.9805	ns
double-D2KD vs. Control	0.9999	ns
double-D2KD vs. Auto-D2KO	0.937	ns
AutoD2 vs. MSN	0.2281	ns
AutoD2 vs. Control	0.2478	ns
AutoD2 vs. Auto-D2KO	0.8453	ns
MSN vs. Control	0.9716	ns
MSN vs. Auto-D2KO	0.8412	ns
Control vs. Auto-D2KO	0.9751	ns

**2W RW ANOVA - Genotype x Phase**			
Interaction (Phase x Genotype)	F (3, 56) = 1.804	0.157	ns
Phase	F (1, 56) = 239.1	<0.0001	****
Genotype	F (3, 56) = 2.643	0.0581	#
**Dunnett's multiple comparison test**			
Dark			
double-D2KD vs. AutoD2		0.0208	*
double-D2KD vs. MSN		0.9934	ns
double-D2KD vs. Control		0.6012	ns
AutoD2 vs. MSN		0.0095	**
AutoD2 vs. Control		0.3323	ns
MSN vs. Control		0.4363	ns
Light			
double-D2KD vs. AutoD2		0.5879	ns
double-D2KD vs. MSN		0.9881	ns
double-D2KD vs. Control		0.9093	ns
AutoD2 vs. MSN		0.7841	ns
AutoD2 vs. Control		0.9329	ns
MSN vs. Control		0.9862	ns

Figure	Measurement	# of mice
S2c	Comparison of controls ($Drd2^{f/w}$, $Drd2^{w/w}$: Dat-cre, A2A-cre, Dat-cre+A2A-cre)<br>Light-Dark Box: Duration in Light Area	n = 5 - 19
S2e-f	Elevated Zero Maze: % Time in open	n = 13 - 38
S3g	Elevated Zero Maze: Number of entries into open	n = 13 - 38

Statistical test	Results	p value	Significance?
**One-way ANOVA**	F (3, 35) = 0.7697	0.5188	ns
**Dunnett's multiple comparisons test**			
control (f/w) vs. control (w/w) (DAT-Cre +/-)		0.5345	ns
control (f/w) vs. control (w/w) (A2a-Cre +/-)		0.9382	ns
control (f/w) vs. control (w/w) (DAT-Cre +/- ; A2a-Cre +/-)		0.9543	ns
**2W ANOVA - Genotype x Age**			
Interaction (Genotype x Age)	F (3, 173) = 1.540	0.206	ns
Genotype	F (3, 173) = 2.982	0.0328	*
Age	F (1, 173) = 83.17	<0.0001	****
**Fishers Least Significant Difference tes**			
12 weeks			
Control vs. double-D2KD		0.2937	ns
Control vs. Auto-D2KD		0.0685	#
Control vs. MSN-D2KD		0.6819	ns
24 weeks			
Control vs. double-D2KD		0.0138	*
Control vs. Auto-D2KD		0.0954	ns
Control vs. MSN-D2KD		0.0532	#
**2W ANOVA - Genotype x Age**			
Interaction (Genotype x Age)	F (3, 173) = 0.6243	0.6002	ns
Genotype	F (3, 173) = 10.14	<0.0001	****
Age	F (1, 173) = 49.37	<0.0001	****
**Fishers Least Significant Difference tes**			
12 weeks			
Control vs. double-D2KD		0.018	#
Control vs. Auto-D2KD		0.8228	ns
Control vs. MSN-D2KD		0.0016	**
24 weeks			
Control vs. double-D2KD		0.0016	**
Control vs. Auto-D2KD		0.2962	ns
Control vs. MSN-D2KD		0.0014	**

Figure	Measurement	# of mice	Statistical test
S3c	15 mg/kg Conditioned Place Preference: Pretest Time on Grid	n = 9 - 10, per contitioned floor, per genotype	**2W ANOVA - Conditioning Floor x Genotype**<br>Interaction (Conditioning Floor x Genotype)<br>Conditioning Floor<br>Genotype
S3c	15 mg/kg Conditioned Place Preference: % Time on Drug-Paired Floor	n = 14 - 20	**2W mixed-effects model - Genotype x Test**<br>Interaction (Test x Genotype)<br>Test<br>Genotype<br>**Dunnett's multiple comparisons test**<br>*Auto-D2KD*<br>Pretest vs. Test 1<br>Pretest vs. Test 2<br>*MSN-D2KD*<br>Pretest vs. Test 1<br>Pretest vs. Test 2<br>*Control*<br>Pretest vs. Test 1<br>Pretest vs. Test 2<br>*Double-D2KD*<br>Pretest vs. Test 1<br>Pretest vs. Test 2

Results	p value	Significance
F (3, 67) = 0.1419	0.9345	ns
F (1, 67) = 0.006484	0.9361	ns
F (3, 67) = 0.1231	0.9461	ns
F (5.785, 123.4) = 0.8684	0.5171	ns
F (1.928, 123.4) = 45.14	<0.0001	****
F (3, 71) = 0.6003	0.6169	ns
	0.0052	**
	0.0456	*
	<0.0001	****
	0.0014	**
	0.0101	*
	0.0016	**
	0.0013	**
	0.0061	**

Figure	Measurement	# of mice	Statistical [illegible]	Results	p value	Significance [illegible]

Figure	Measurement	# of mice	Statistical test	Results	p value	Significance?
